# Enhancement of efficiency in organic photovoltaic devices containing self-complementary hydrogen-bonding domains

**DOI:** 10.3762/bjoc.9.122

**Published:** 2013-06-06

**Authors:** Rohan J Kumar, Jegadesan Subbiah, Andrew B Holmes

**Affiliations:** 1 School of Chemistry, Bio21 Institute, University of Melbourne, VIC 3010, Australia; 2 CSIRO Materials Science and Engineering, Private Bag 10, Clayton South, Victoria 3169, Australia

**Keywords:** hydrogen-bonding, organic photovoltaics, self-assembly

## Abstract

Self-complementary hydrogen-bonding domains were incorporated as the electron deficient unit in “push–pull”, p-type small molecules for organic photovoltaic active layers. Such compounds were found to enhance the fill factor, compared with similar non-self-organized compounds reported in the literature, leading to higher device efficiencies. Evidence is presented that the ability of these molecules to form one-dimensional hydrogen-bonded chains and subsequently exhibit hierarchical self-assembly into nanostructured domains can be correlated with improved device efficiency.

## Introduction

The efficient generation of energy without producing environmentally detrimental side-products is an ongoing challenge for science [1–2]. Incident solar radiation offers the largest source of energy [1]; however, efficient photovoltaic devices with low manufacturing costs and rapid energy payback times are required [3]. The use of organic materials in photovoltaic devices is attractive owing to the abundance of the elements used, the possibility to use low-cost manufacturing techniques, and the diversity of molecular structure that is accessible. There is a growing understanding of the control of hierarchical interactions to produce functional behaviour in molecular materials [4–8]; however, much remains to be investigated. In particular, designed supramolecular interactions that control the spatial and temporal distribution of materials are of interest [9–12].

Polymeric donor materials in blends with solution-processable fullerenes have produced the highest efficiency organic photovoltaic bulk heterojunction (BHJ) devices to date [13–14]. The critical role of supramolecular interactions together with material behaviour at interfaces has been significant in achieving these high efficiencies, as has been highlighted by the use of thermal annealing and solvent additives to produce dramatic efficiency increases [15]. In their role as systems for studying supramolecular interactions, polymers suffer from the added complexity of compositional factors such as molecular weight and polydispersity [16–17], as well as the technical difficulty of studying morphology changes in thin films. Whilst high-efficiency BHJ devices based on solution-processed small molecules have also relied on the use of solvent additives in order to achieve high efficiencies [18–19], they offer a relatively simple basis for designing supramolecular interactions. Such interactions can be readily investigated in solution and the results can be translated to the solid state. As such, these supramolecular interactions may offer a means to control bulk morphology without reliance on post-processing, leading to cost and energy input advantages in device manufacture.

**Figure 1:**
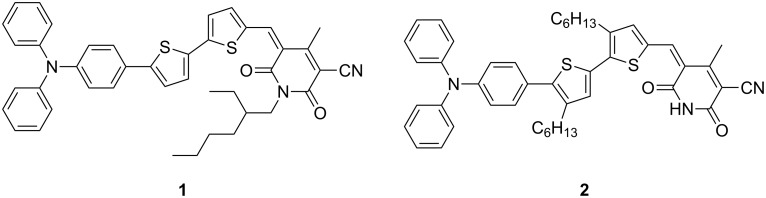
*N*-ethylhexyl-substituted (**1**) [20] and target free N–H (**2**) cyanopyridone structures.

Recently, a donor–acceptor small molecule with a cyanopyridone moiety as the acceptor motif and displaying moderate photovoltaic efficiency in a BHJ device with a fullerene was reported [20]. The efficiency of these optimized devices was primarily limited owing to a low fill factor, which was attributed to poorly interconnected domains for charge transport in the cyanopyridone. The *N*-2-ethylhexyl substituent was employed to enhance the solubility of compound **1** in organic solvents (Figure 1). However, alkylation on the nitrogen masks a known self-complementary hydrogen-bonding domain. The self-assembly properties of this unit have been extensively studied in a variety of systems [21–26], but this approach has not yet been applied to improving organic photovoltaic device performance. Solubility could be maintained by introduction of alkyl substituents at the β-position of the thiophenes, and the NH-cyanopyridone unit could be unmasked for hydrogen-bonding. In turn, this may allow access to higher order supramolecular structures, promoting charge transport and increasing the fill factor and efficiency of devices based on this compound.

## Results and Discussion

### Synthesis

**Scheme 1:**
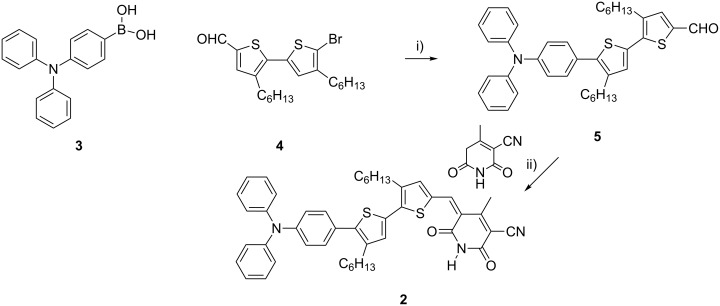
Synthesis of the cyanopyridone **2**, reagents and conditions: (i) Pd(PPh_3_)_4_, Cs_2_CO_3_, toluene, reflux, 16 h, 84%; (ii) EtOH, reflux, 2 h, 82%.

The synthesis of compound **2** was completed in a simple two-step procedure by using the strategy of Gupta et al. [20] (Scheme 1). A Suzuki coupling of the triarylaminoboronic acid **3** to the bromo-dihexylbithiophene-carboxaldehyde **4** [27] followed by a Knoevenagel condensation provided the target compound **2** in excellent yield. The solubilizing strategy of incorporating alkyl groups on the thiophene unit provided the compound with excellent solubility in a wide range of common solvents.

### Optoelectronic properties

**Figure 2:**
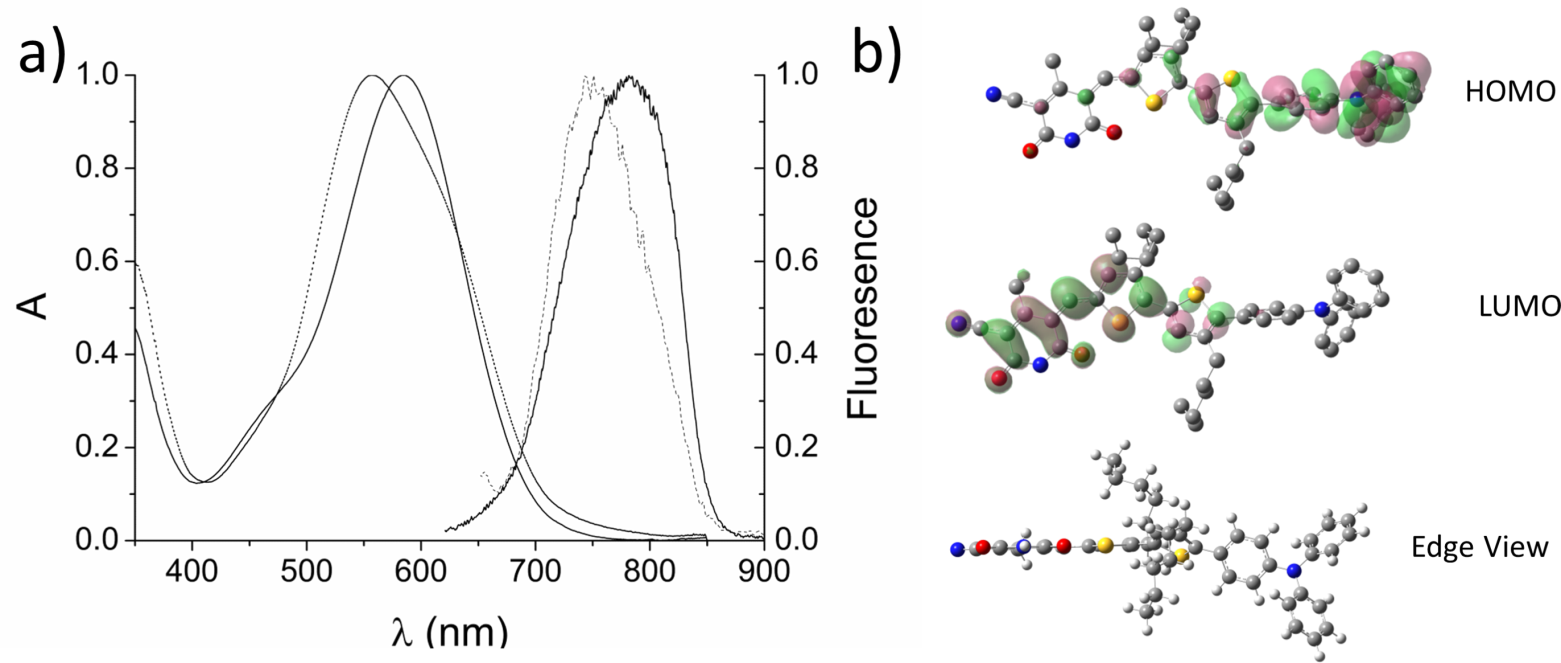
(a) Chloroform (solid line) and thin-film (dashed line) UV–vis absorption and emission spectra and (b) optimized DFT structure and calculated orbital densities of compound **2**.

In chloroform solution the UV–vis absorption spectrum of compound **2** exhibited a peak absorbance of 586 nm (Figure 2a), compared with 590 nm for the N-alkylated analogue **1**. This transition is assigned to an intramolecular charge-transfer state from the electron-rich triarylamine moiety to the electron-deficient cyanopyridone. The absorption peak of **2** displays a 30 nm hypsochromic shift in the solid state, with the maximum absorption at 556 nm, and the absorption is also broadened with a shoulder at approximately 614 nm. When compound **2** in chloroform solution is excited at the absorption maximum, the emission spectrum shows a weak peak at 782 nm. The emission maximum of a thin film of **2** is hypsochromically shifted by 31 nm to 751 nm. Cyclic voltammetry and photoelectron spectroscopy in air (PESA) were used to determine the HOMO and LUMO levels of compound **2** and these are summarized in Table 1. These values are similar to those reported for compound **1** [20] and are suitable for charge transfer to take place to fullerene-based electron acceptors, such as PC_61_BM. The NH cyanopyridone unit can presumably exist as a number of tautomers; however, if present, these do not appear to affect the electronic properties in solution significantly compared with the N-alkylated compound.

**Table 1:** Optoelectronic and electrochemical (CV) data for compounds **1** and **2**.

	Absorption (nm)		Emission (nm)		*E*_½_^a^(Δ*E*_p_)^b^ mV			HOMO^c^ (eV)	*E*_g_^d^ (eV)	LUMO^e^ (eV)
	λ_max_ (CHCl_3_)	λ_max_ (film)	λ_max_ (CHCl_3_)	λ_max_ (film)	R/R^−^	R^+^/R	R^2+^/R			

**1**^f^	590	584	800	—	—	—	—	−5.4	1.6	−3.8
**2**	586	556	782	751	−1136^g^	464 (60)	786 (70)	−5.26 (−5.55)	1.6 (1.5)	−3.66 (−4.05)

^a^Half wave potential determined as the average of the anodic and cathodic peak potentials, *V* versus ferrocene 10 mM in CH_2_Cl_2_, 0.1 M Bu_4_N(PF_6_). ^b^Difference between the anodic and cathodic peak potentials, *V*, v = 0.1 mV s^−1^. ^c^Determined from *E*_HOMO_ = −(*E*_ox_ + 4.80) eV, data in brackets measured by PESA on thin films. ^d^Determined from the difference in electrochemically measured HOMO and LUMO, data in brackets estimated from the onset of absorption in thin films. ^e^Determined from *E*_LUMO_ = −(*E*_red_ + 4.80) eV, data in brackets measured by PESA on thin films and absorption onset *E*_LUMO_ = *E*_HOMO_ + *E*_g_. ^f^Data from [20]. ^g^Irreversible reduction, value in the peak reduction potential.

Density functional theory (DFT) calculations, at the B3YLP-61G*(d,p) level of theory using the Gaussian ‘09 suite of programs [28], result in the observation of a polar distribution of the calculated molecular orbital densities for **2**, as expected from the dipolar absorption transitions observed (Figure 2b). A conformational search followed by DFT optimization of the resultant structures showed that the monomer has an energetic preference for the flat structure shown in Figure 2b, with a barrier to rotation for the thiophene alkene bond of 272 KJ mol^−1^. This is supported by the crystal structure of a related NH-cyanopyridone structure determined by Würthner et al. [29], which exhibited a similarly oriented, flat thiophene cyanopyridone structure in a hydrogen-bonded dimer. The barrier for rotation, as determined by temperature-dependent ^1^H NMR analysis, was reported to be at least 60 KJ mol^−1^ [29].

### Self-assembly of cyanopyridone **2**

**Figure 3:**
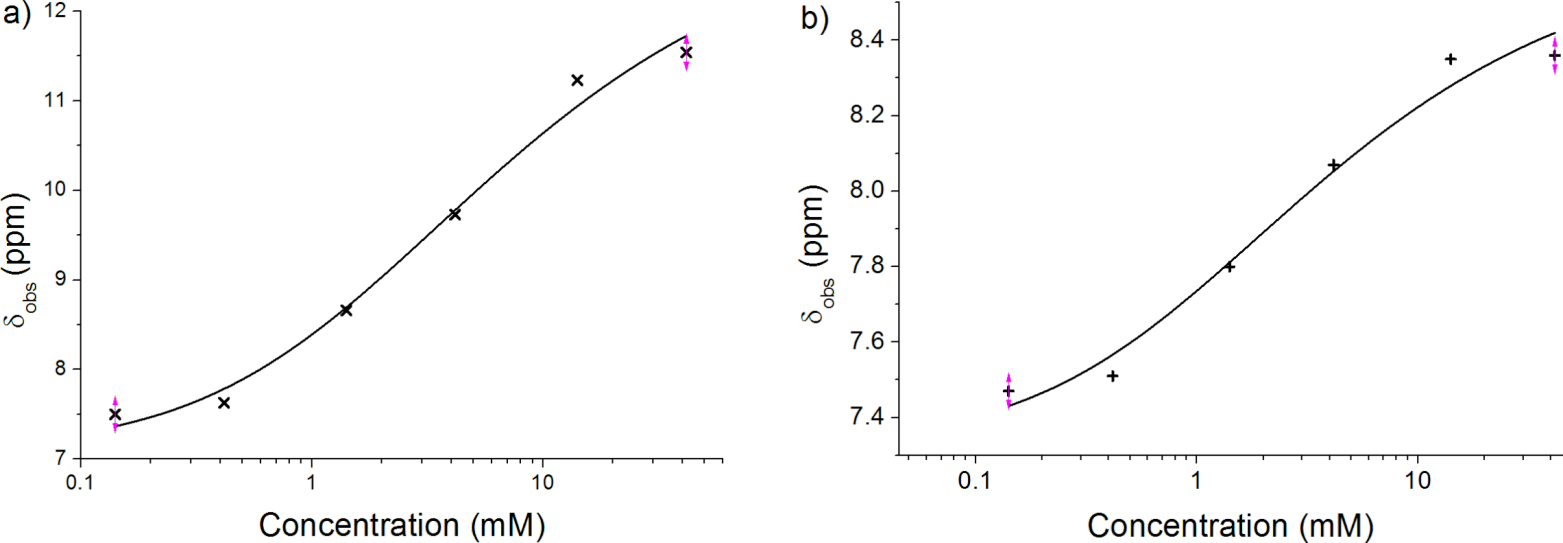
The concentration dependence of (a) the NH ^1^H NMR chemical shift and (b) the vinyl proton chemical shift for compound **2** in *d*_6_-benzene. The line is the regression fit of Equation 1 to the data.

The hydrogen-bond-mediated self-assembly behaviour of compound **2** was monitored by ^1^H NMR spectroscopy. The chemical shift of the proton on nitrogen in chloroform was observed to shift downfield in a concentration-dependent manner. Regression analysis of the concentration-dependent equilibrium was not possible in chloroform as the maximum ^1^H chemical shift was not reached at concentrations as high as 30 mg/mL (see Supporting Information File 1). However, in *d*_6_-benzene observation of the maximum and minimum chemical shift of the NH proton was possible in an accessible concentration range (Figure 3a). It was also observed that other chemical shifts within the molecule exhibit a concomitant concentration-dependent downfield shift, notably the vinyl proton (Figure 3b). These experiments were replicated in the presence of an excess amount of PC_61_BM and no significant difference in concentration-dependent change of chemical shift was observed (see Supporting Information File 1).

The variation of chemical shift with concentration for both the cyanopyridone NH and the vinyl proton could be fitted by a theoretical expression for dimerization derived from a two-state equilibrium (Equation 1, fit shown in Figure 3) [25]. It should be noted that Equation 1 describes the simplest two-state model of association, namely dimerization, but can also be used to fit a two-state isodesmic self-association interaction; the two cases are indistinguishable using this model [30]. Crystal structures and scanning tunnelling microscopy studies of related compounds show that both dimers [29], higher order *n*-mers [31], and chains of hydrogen-bonded molecules are possible [32].

[1]
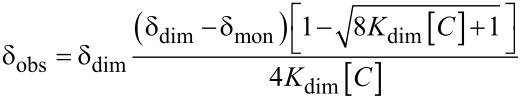


**Figure 4:**
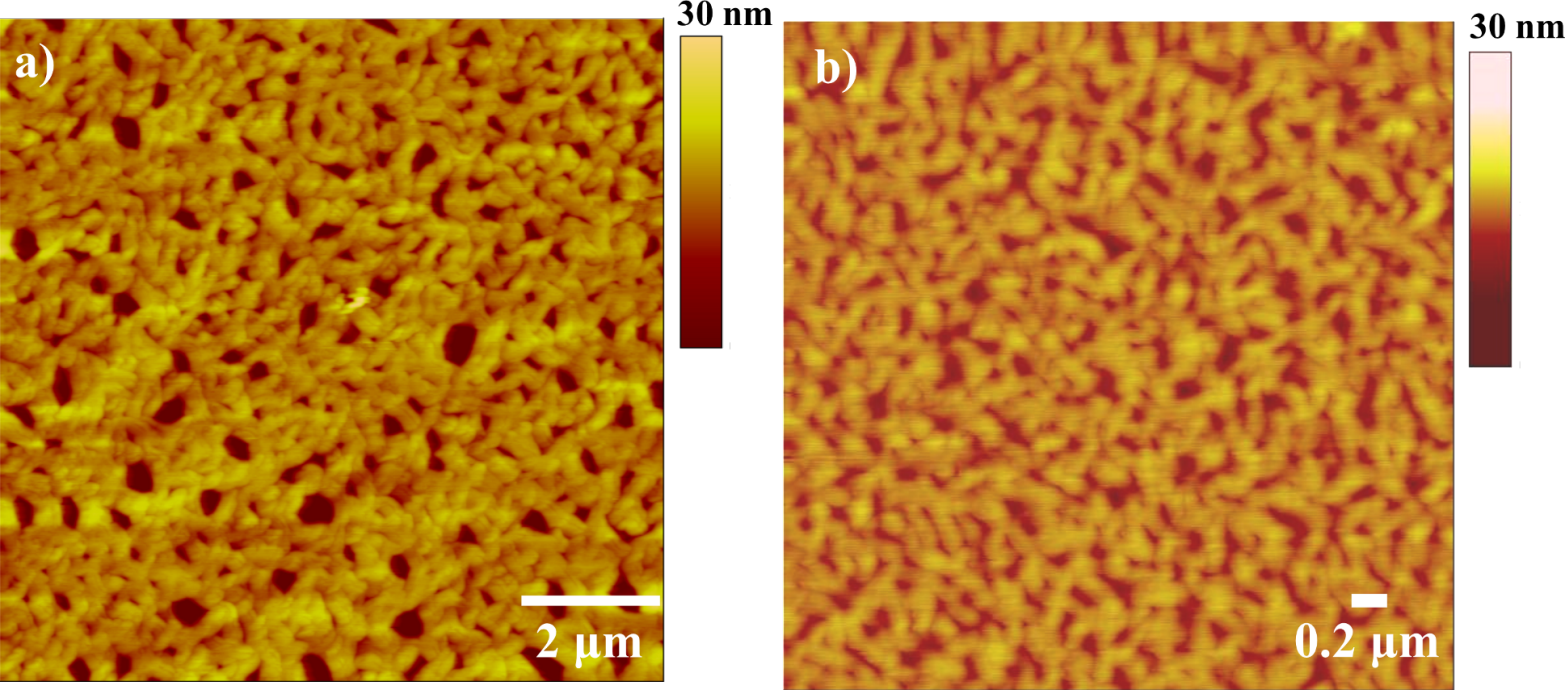
AFM height image of observed nanostructures of films drop cast from (a) 10 mg/mL and (b) 1 mg/mL chloroform solution of **2**.

An additional observation from the ^1^H NMR experiments was gel formation at the highest concentrations, indicating the formation of high-aspect-ratio nanofibers in solution [33]. Films drop cast from 1 mg/mL and 10 mg/mL solutions of **2** in chloroform were investigated by AFM in order to directly observe any nanostructures present. A representative scan at each concentration is shown in Figure 4. The films were found to contain interconnected fibrous nanostructures approximately 85–110 nm in width at both concentrations; however, the structures appeared to be more regular in films cast from solutions at higher concentrations.

Hydrogen-bond-mediated self-association of molecules bearing a self-complementary imide motif has been well studied by ^1^H NMR, as reported in the literature [21–26]. Given this precedent, the observed concentration-dependent downfield change in the NH chemical shift is assigned to intermolecular hydrogen bonding. The downfield chemical shift of other protons in molecules bearing a cyanopyridone motif is unprecedented and at least three scenarios may be invoked to explain this observation. Since the ^1^H chemical shift data can support either a dimerization or isodesmic self-association model as the initial self-assembly step, both models must be considered.

**Figure 5:**
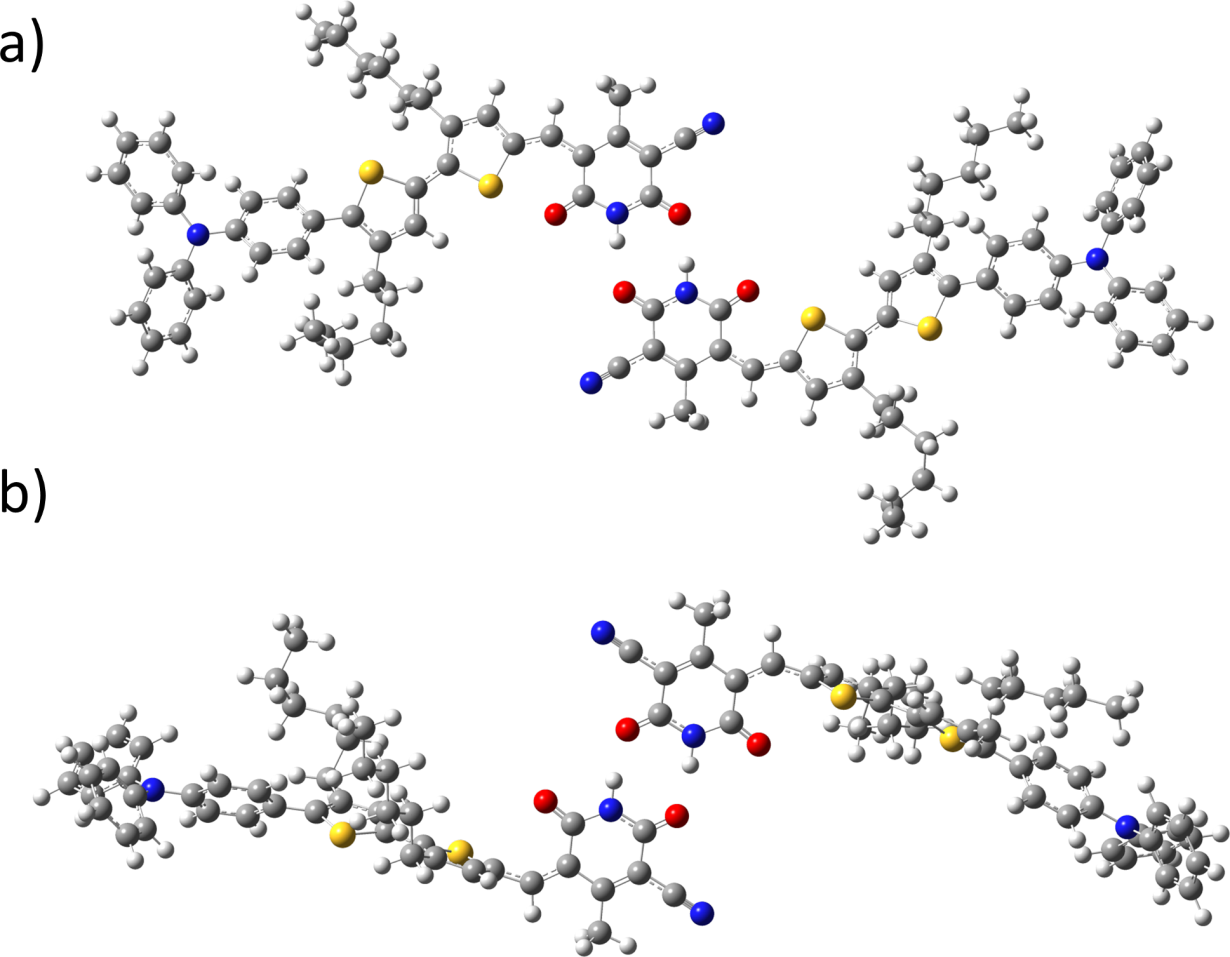
Dimeric structures of **2**. (a) centrosymmetric dimer of **2**; (b) bond-rotated centrosymmetric dimer of **2** as predicted by DFT calculations.

The hydrogen-bond-mediated centrosymmetric dimerization of two molecules of **2**, leading to an alteration of the charge distribution and thus the ^1^H chemical shift of a number of protons is the simplest explanation of the NMR data (Figure 5). This does not explain the formation of nanostructures as observed by AFM and the gelation behaviour at high concentration. Alternatively, isodesmic self-association to form one dimensional hydrogen-bonded chains of **2** may incur conformational changes in **2** for steric reasons, which could result in changes to the ^1^H chemical shifts of **2**. The final scenario considered here is that the hydrogen-bonding-mediated interaction is immediately followed by a kinetically faster second supramolecular interaction, leading to the observation by ^1^H NMR of only the rate-limiting first step. π–π stacking interactions are observed for planar aromatic systems and dimerized nucleobases; however, in these cases an upfield change in chemical shift is generally observed, [5,30] in contrast to the downfield movement in chemical shift observed for the vinyl proton in compound **2**. Non-specific van der Waals interactions are typically weaker than hydrogen-bonding interactions and insufficient to explain the changes in chemical shift at the concentrations observed here. However, given the strongly dipolar nature of the compound, the role of dipole-based interactions cannot be ruled out. We then turned to molecular modelling to shed further light on the possible modes of self-organization.

In order to further investigate the plausibility of each scenario, GIAO shielding calculations were used to predict the ^1^H NMR chemical shifts of (i) the optimized structure of **1** (Figure 2), (ii) an *anti*-symmetric dimer of **2** (Figure 5a) and (iii) the same dimer structure in which the C–C single bond connecting the alkene to the adjacent thiophene ring is rotated by 90° (Figure 5b). This bond rotation is the major conformational change possible for **2** in order to accommodate the steric bulk of the molecule in hydrogen-bonded chains. It is also likely to produce significant changes in the alkene proton chemical shift. These calculated chemical shifts are compared with those obtained experimentally (Table 2).

**Table 2:** Experimental versus calculated proton chemical shifts in the dimers shown in Figure 5.^a^

Condition	NH proton chemical shift (ppm)	alkene proton chemical shift (ppm)

0.14 mM **2** in *d*_6_-benzene	7.50	7.47
42 mM **2** in *d*_6_-benzene	11.54	8.36
**2** in Figure 2 (calculated)	6.92	7.50
Optimized dimer of **2** in Figure 5a (calculated)	11.06, 10.97	7.45, 7.48
Bond-rotated dimer of **2** in Figure 5b (calculated)	10.79, 10.70	8.51, 8.24

^a^DFT NMR calculations where performed using the Gaussian 09 suite of programs and the BYLP 631G(d) basis set. Solvation was not considered.

The calculated ^1^H chemical shifts for the optimized structure of **2** are in close agreement with the experimentally observed values at low concentration. This close agreement between experimental observation and calculated value is observed for both the NH proton resonance (δ 7.50 versus 6.92, respectively) and the alkene proton resonance (δ 7.47 and 7.50, respectively). The calculated ^1^H NMR chemical shifts for the optimized dimer of compound **2** shows a large downfield shift (Δδ 4.14 ppm) for the NH proton resonance, which is in approximate agreement with the experimental value (Δδ 4.04 ppm), and a small upfield change in the chemical shift of the alkene proton resonance (Δδ 0.05 ppm) in contrast with the downfield change observed (Δδ 0.89 ppm). Bond rotation of the alkene thiophene bond and subsequent calculation of the ^1^H NMR chemical shifts gives a downfield change in chemical shift for the same proton (Δδ 1.01 ppm), in agreement with experimental observations (Δδ 0.89 ppm). Thus, of the scenarios considered, that which best fits the data is hydrogen bonding of the NH proton with a concomitant rotation of the alkene thiophene bond. Due to the empirically demonstrated barrier to bond rotation for these compounds [20], this implies the need to accommodate the steric bulk of the molecule, possibly in extended one-dimensional hydrogen-bonded arrays formed under isodesmic kinetic control.

**Figure 6:**
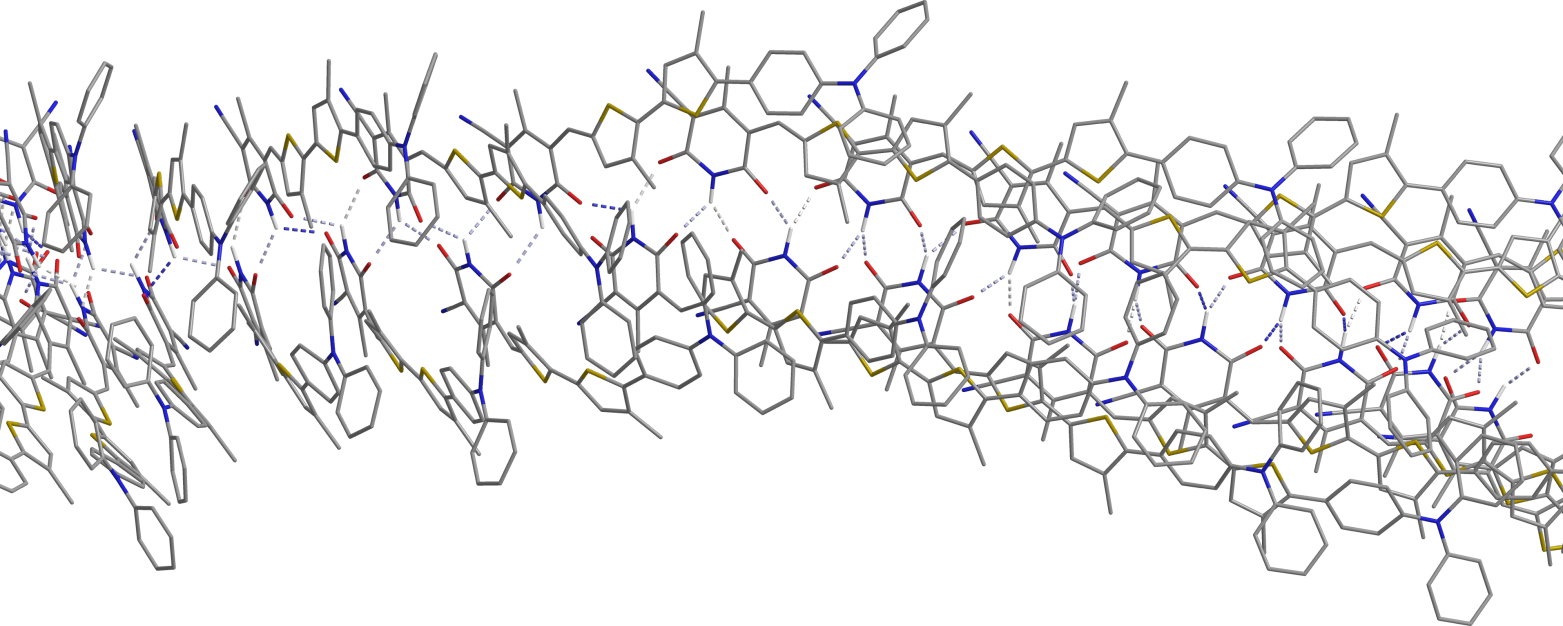
Schematic representation of self-complementary interactions leading to one-dimensional chains.

The formation of interconnected nanostructures by **2**, as observed in the AFM experiments, also leads to the interpretation of the ^1^H NMR self-association data as the formation of one-dimensional chains of **2**. One possible mode of self-complementary interaction leading to one-dimensional chains is represented in Figure 6. The nanostructures formed are in the region of 85–100 nm in size and this is an order of magnitude larger than the width of two molecules (8 nm). The UV–vis absorption spectrum of a thin film of **2** shows an asymmetric hypsochromic shift of the absorption peak (Figure 2a) indicative of the formation of weak H-aggregates in the solid state [34–35]. Thus, a hierarchical model for association can be developed in which the formation of hydrogen-bonded chains of **2** is followed by interchain H-aggregation, although this requires further experimental verification.

### Organic photovoltaic device performance

Finally, organic photovoltaic (OPV) BHJ devices with active layers comprising 1:1 mixtures of **2** and PC_61_BM spin-coated from chloroform solutions of various concentrations were fabricated in order to determine the functional consequences of the observed self-assembly behaviour. The architecture of the devices was glass/ITO/poly(3,4-ethylenedioxythiophene)poly(styrenesulfonate)/active layer/ZnO nanoparticles/Al. Zinc oxide nanoparticles were used as an electron transport layer. The spin-coating velocity was varied to control film thickness. The average results from ten devices are summarized in Table 3 and the devices show significant performance variation with concentration. The open-circuit voltage (*V*_oc_) varies between 0.90 and 0.96 for the four conditions tested. The fill factor (FF) varies from 49% to 37% and the short circuit current (*J*_sc_) varies between 5.1 and 3.05 mA/cm^2^. The devices fabricated from 10 mg/mL, 1:1 solutions of **2**:PC_61_BM show the highest efficiency and also the highest *V*_oc_, FF and *J*_sc_.

**Table 3:** OPV device performance.^a^

Compound	Concentration (mg/mL)^b^	*J*_sc_ (mA/cm^2^)^c^	*V*_oc_ (V)^d^	FF (%)^e^	η(%)^f^

**2**	6	4.40 (4.55)	0.94 (0.94)	48 (49)	2.02 (2.10)
	10	5.10 (5.30)	0.96 (0.96)	49 (51)	2.40 (2.58)
	15	3.90 (4.00)	0.92 (0.92)	40 (41)	1.42 (1.50)
	20	3.05 (3.20)	0.90 (0.90)	37 (38)	1.02 (1.10)
**1**^g^	20	6.77	0.87	38	2.25

^a^Data are the mean for *n* = 10 devices, and best device data is shown in brackets. ^b^The active layers were spin coated from 1:1 (w/w) solutions of **2**:PC_61_BM at the concentrations shown. ^c^Short circuit current. ^d^Open circuit voltage. ^e^Fill Factor. ^f^Device energy conversion efficiency (AM 1.5). ^g^Data is from the best device.

A primary observation is that optimized devices using compound **2** exhibit marginally higher average performances than any of the devices reported by Gupta et al. using the N-alkylated compound **1** (2.40% versus 2.25% when **1** was deposited from chlorobenzene and 1.64% when **1** was deposited from chloroform) [20]. The primary parameter for which compound **2** outperforms its N-alkylated analogue **1** is the fill factor. Fill factor is believed to be optimized when charge conduction to the electrodes is optimized, and compounds that self-assemble into arrays have been observed to display high fill factors [36]. Thus, the formation of one-dimensional chains of **2** via hydrogen bonding may create networks that facilitate hole percolation to the electrodes, resulting in an increase in fill factor [37].

The variation of performance with the concentration of the solutions used to deposit the active layer gives insight into the correlation of supramolecular interaction and functional performance. It is seen that the performance of the devices increased with the concentration of active layer components in the deposition solution, (e.g., 6 mg/mL and 10 mg/mL) and the efficiency dropped when deposition was carried out from solutions at higher concentration of the active layer. This was primarily due to the poor film-forming ability of the more concentrated solutions as they approached the concentration at which organogel formation occurred. This is a similar effect to that observed for highly crystalline materials [38], for which performance drops rapidly as the crystal domains exceed a certain size. For compound **2**, the supramolecular interaction can be quantified in a variety of solvents and the domain size controlled through the judicious choice of solvent. This will be advantageous for use in high-throughput production methods, ultimately lowering the cost and energy for the manufacture of organic photovoltaic devices.

## Conclusion

A self-complementary hydrogen-bonding domain has been incorporated as the electron-deficient moiety in a “push–pull” p-type molecule **2** for organic solar cells. Self-association of this domain could be monitored by ^1^H NMR, and at higher concentrations the compound was found to form nanostructures and organogels. In bulk heterojunction organic photovoltaic devices fabricated with 1:1 blends of **2** with PCBM it was observed that the fill factor increased in comparison with the data reported for a similar molecule (**1**) that did not show evidence of self-organization. The use of designed hydrogen-bonding interactions to produce hierarchical materials may be a suitable approach to improve OPV device efficiencies through the enhanced self-organization of materials.

## Supporting Information

File 1 Full experimental details and compound characterization.
