# Supplementary material for: Enhancement of efficiency in organic photovoltaic devices containing self-complementary hydrogen-bonding domains
Source: Beilstein J Org Chem. 2013 Jun 6;9:1102–10. doi: 10.3762/bjoc.9.122 (PMC3701423; doi:10.3762/bjoc.9.122)
Supplement: File 1 — Full experimental details and compound characterization. [file Beilstein_J_Org_Chem-09-1102-s001.pdf]

Supporting Information

for

Enhancement of efficiency in organic photovoltaic devices containing self-complementary hydrogen-bonding domains

Rohan J. Kumar*[1,2], Jegadesan Subbiah[1] and Andrew B. Holmes[1,2]

Address: [1]School of Chemistry, Bio21 Institute, University of Melbourne, VIC 3010, Australia and [2]CSIRO Materials Science and Engineering, Private Bag 10, Clayton South, Victoria 3169, Australia

Email: Rohan J. Kumar - jkumar@unimelb.edu.au

* Corresponding author

Full experimental details and compound characterization

Synthetic chemistry

Dry tetrahydrofuran (THF), CH_2Cl_2, and toluene were obtained by passing these solvents through activated alumina columns. 5'-Bromo-3,4'-dihexyl-[2,2'-bithiophene]-5-carbaldehyde was prepared as described in [1] and 4-methyl-2,6-dioxo-1,2,5,6-tetrahydropyridine-3-carbonitrile was prepared as described in [2]. All other reagents were purchased from Sigma-Aldrich and used as received. Analytical thin-layer chromatography (TLC) was conducted on Sigma-Aldrich silica-gel-coated aluminium sheets and visualised with UV. Flash chromatography was carried out using Merck Kieselgel 60 (230–400 mesh; particle size 0.04–0.63 mm) silica gel.

Analytical chemistry

^{1}H and ^{13}C NMR spectra were recorded on a Bruker Av400 spectrometer at 400 MHz and 100.6 MHz, respectively. Chemical shifts (δ) are measured in ppm and referenced internally to the residual solvent signal. Melting points were recorded on an Electrothermal IA9300 digital melting point apparatus. Positive ion EI mass spectra were run on a ThermoQuest MAT95XL mass spectrometer using an ionization energy of 70 eV. Accurate mass measurements were obtained with a resolution of 5000–10000 using perfluorokerosene as the reference compound. Microanalyses were performed by Chemical and MicroAnalytical Services Pty. Ltd. Belmont, 3216, Australia. The AFM samples were prepared by drop casting compounds (1 mg/mL in chloroform) on a Silicon substrate. Tapping-mode AFM (NanoScope II, Dimension, Digital Instrument Inc.) was carried out with commercially available tapping-mode tips.

Synthetic procedures and characterization

5'-(4-(Diphenylamino)phenyl)-3,4'-dihexyl-[2,2'-bithiophene]-5-carbaldehyde (**5**)

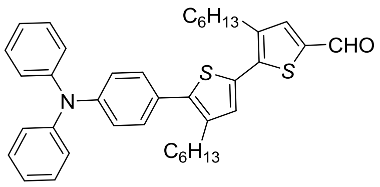

A solution of (4-(diphenylamino)phenyl)boronic acid (**3**, 0.50 g, 1.7 mmol), 5'-bromo-3,4'-dihexyl-[2,2'-bithiophene]-5-carbaldehyde (**4**, 0.69 g, 1.6 mmol) and Cs_2CO_3 (1.537 g, 4.72 mmol) in toluene (20 mL) was degassed for 10 min. $Pd(PPh_3)_4$ (0.091 g, 0.079 mmol) was added and the resulting solution was heated under reflux for 16 h under inert atmosphere. The solution was cooled to room temperature and diluted with EtOAc, filtered and concentrated in vacuo. The resulting crude residue was purified by flash chromatography (SiO_2, *n*-heptane, 5% EtOAc) to give **5** as a bright orange oil (0.80 g, 1.3 mmol, 84%). R_f 0.24 (*n*-heptane, 5% EtOAc); IR (neat) 2953, 2924, 2854, 1662, 1589, 1532, 1506, 1492, 1454, 1397, 1376, 1368, 1325, 1314, 1274, 1239, 1192, 1177, 1152, 1116, 1088, 1076, 1028, 908, 834, 826, 752, 730, 694, 671, 648, 637, 622 cm^{-1}; ^1H NMR (400 MHz, $CDCl_3$) δ 9.80 (s, 1H), 7.57 (s, 1H), 7.33–7.21 (m, 6H), 7.18–7.02 (m, 9H), 2.85–2.79 (m, 2H), 2.70–2.64 (m, 2H), 1.75–1.53 (m, 4H), 1.51–1.19 (m, 12H), 0.97–0.80 (m, 6H); ^{13}C NMR (101 MHz, $CDCl_3$) δ 182.5, 147.7, 147.5, 142.0, 140.4, 140.0, 139.9, 139.19, 139.16, 132.4, 130.2, 130.0, 129.5, 127.5, 124.9, 123.5, 122.9, 31.8, 31.0, 30.4, 29.6, 29.3, 29.2, 28.9, 22.7, 14.2; LRMS (EI) *m*/*z* (%): 168 (10), 605 (100), 606 (42), 607 (17); HRMS–EI (*m*/*z*): $[M]^{+\bullet}$ calcd for $C_{39}H_{43}NO^{32}S_2$: 605.2786, found 605.2778.

(*Z*)-5-((5'-(4-(Diphenylamino)phenyl)-3,4'-dihexyl-[2,2'-bithiophen]-5-yl)methylene)-4-methyl-2,6-dioxo-1,2,5,6-tetrahydropyridine-3-carbonitrile (**2**)

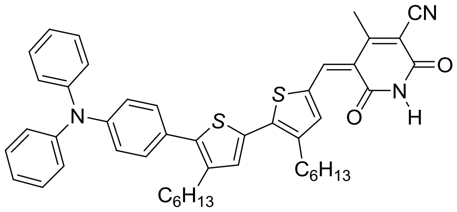

A solution of **5** (0.10 g, 0.17 mmol) and 4-methyl-2,6-dioxo-1,2,5,6-tetrahydropyridine-3-carbonitrile (0.038 g, 0.26 mmol) was refluxed in EtOH (10 mL) for 2 h. The solution was cooled to room temperature and the precipitate collected by filtration. The precipitate was then washed with ethanol and heptane to give **2** (0.10 g, 0.14 mmol, 82% yield) as a dark purple solid. Mp 222–225 °C; R_f 0.63 (CH_2Cl_2, 5% CH_3OH); IR (neat) 2954, 2926, 2853, 2219, 1674, 1622, 1593, 1574, 1534, 1494, 1455, 1412, 1378, 1345, 1333, 1303, 1282, 1263, 1189, 1178, 1167, 1089, 1067, 1052, 1029, 1016, 783, 753, 696, 613 cm^{-1}; ^1H NMR (400 MHz, $CDCl_3$) δ 8.20 (s, 1H), 7.88 (s, 1H), 7.63 (s, 1H), 7.46 (s, 1H), 7.36–7.27 (m, 6H), 7.18–7.04 (m, 8H), 2.91–2.84 (m, 2H), 2.72–2.63 (m, 5H), 1.78–1.60 (m, 4H), 1.50–1.22 (m, 12H), 0.94–0.85 (m, 6H); ^{13}C NMR(126 MHz, $CDCl_3$) δ 162.9, 160.9, 160.6, 152.6, 149.8, 148.0, 147.4, 144.5, 143.3, 140.6, 140.1, 134.3, 132.3, 132.1, 129.9, 129.6, 127.0, 125.1, 123.7, 122.7, 115.0, 114.9, 31.8, 31.8, 31.1, 30.0, 29.6, 29.4, 29.3, 28.9, 22.7, 19.4, 14.3, 14.2.; LRMS (EI): *m*/*z* (%): 55 (11), 57 (89), 69 (49), 71 (61), 83 (66), 85 (22), 95 (45), 97 (73), 111 (29), 737 (100), 738 (39); HRMS (EI) Calcd for $C_{46}H_{47}N_3O_2{}^{32}S_2$: 737.3110 $[M]^{+\bullet}$, found 737.3089. Anal. calcd for $C_{46}H_{47}N_3O_2{}^{32}S_2$: C, 74.9; H, 6.4; N, 5.7; found: C, 75.1; H, 6.45; N, 5.3.

Synthesis of ZnO nanoparticles

Zinc oxide (ZnO) nanoparticles were synthesized as reported [3] by a sol–gel method using precursors of $Zn(CH_3COO)_2 \cdot 2H_2O$ and $(CH_3)_4NOH$ (TMAH). In a typical process, ZnO nanoparticles are synthesized by dropwise addition of 0.4 M TMAH dissolved in ethanol to 0.1 M $Zn(CH_3COO)_2 \cdot 2H_2O$ dissolved in dimethyl sulfoxide (DMSO). The resulting ZnO nanoparticles are washed and dispersed in ethanol at 25 mg/mL.

Device fabrication

Bulk-heterojunction (BHJ) solar cells were fabricated by the spin coating of 30 nm thick layers of poly(3,4-ethylenedioxythiophene):poly(styrenesulfonate) (PEDOT:PSS; Baytron AI 4083 from HC Starck) on patterned glass substrates, which were washed by acetone, and 2-propanol in an ultrasonication bath and UV/ozone-treated. The PEDOT:PSS films were baked at 140 °C for 10 min in air. An active layer of the device consisting of the blend of organic donor molecule and $PC_{60}BM$ (99.5% pure, Solenne BV) with a ratio of 1:1 was then spin coated from chloroform solvent with a thickness of ~80 nm. A thin layer of ZnO nanoparticles was deposited on the active layer by spin-coating (3000 rpm) to form 25 nm of a ZnO layer. The films were transferred to a metal evaporation chamber, and aluminum (100 nm) was deposited through a shadow mask (active area was 0.06 cm^2) at approximately 1×10^{-6} torr. Film thickness was determined by Veeco Dektak 150+Surface Profiler. The current density–voltage measurements of the devices were carried out using a 1 kW Oriel solar simulator with an AM 1.5G filter as the light source in conjunction with a Keithley 2400 source

measurement unit. Solar measurements were carried out under 1000 W/m^2 AM 1.5G illumination conditions. For accurate measurement, the light intensity was calibrated by using a reference silicon solar cell (PVmeasurements Inc.) certified by the National Renewable Energy Laboratory. Device fabrication and characterizations were performed in an ambient environment without any encapsulation.

Additional figures

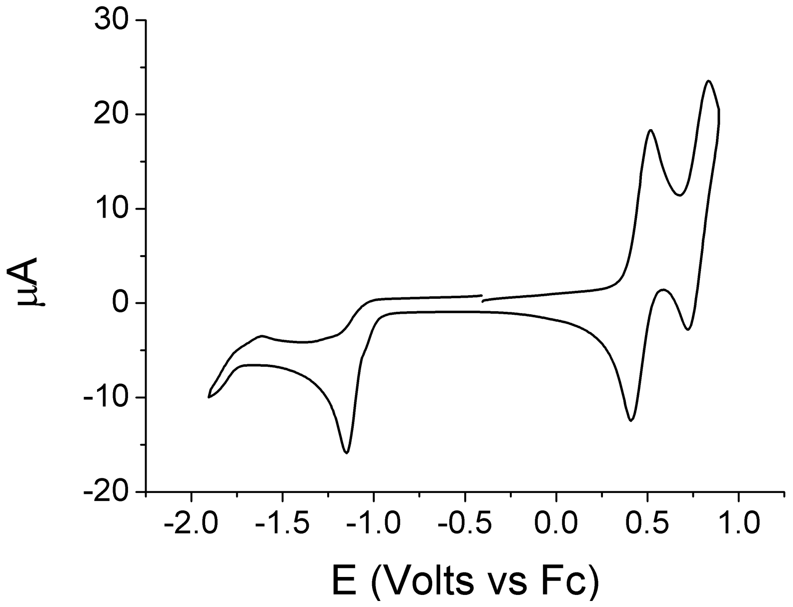

Figure S1: Cyclic voltammetry trace for compound **2**.

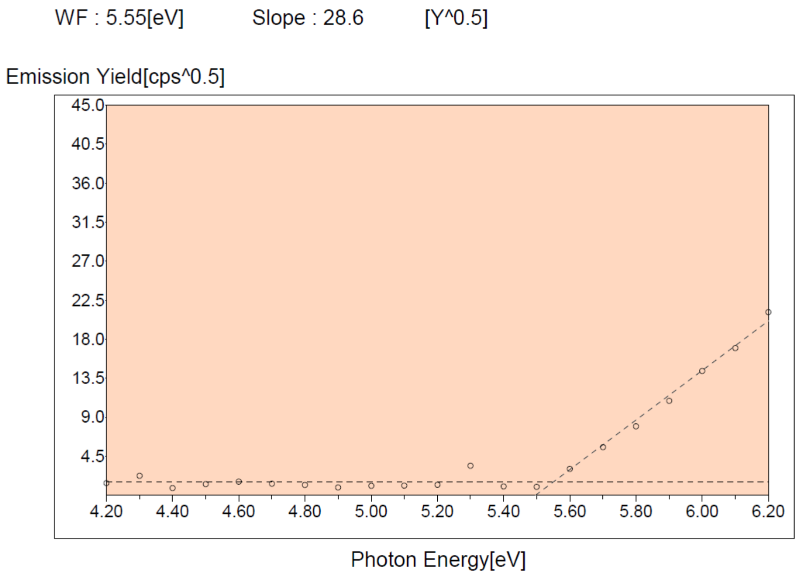

Figure S2: PESA trace for compound **2**.

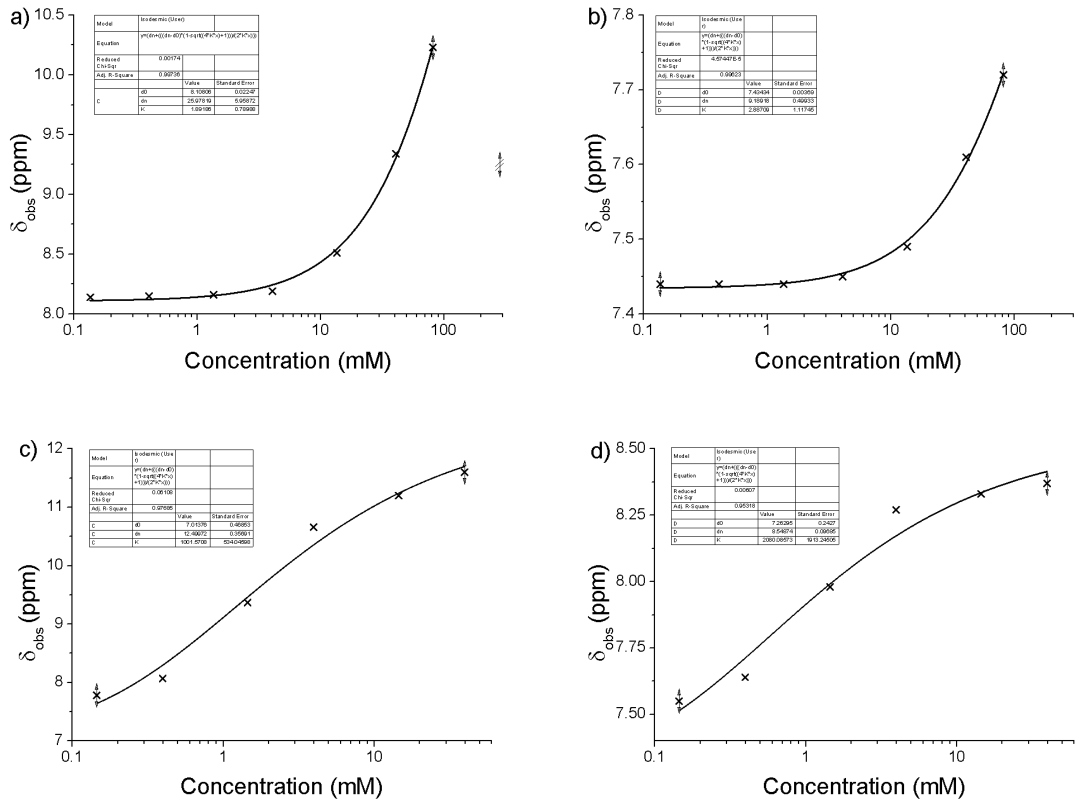

Figure S3: Variation of ^{1}H chemical shift for compound **2**. (a) N proton in $CHCl_3$; (b) alkene proton in $CHCl_3$; (b) N proton 1:1, **2**:PCBM in *d*$_6$-benzene; (d) alkene proton 1:1, **2**:PCBM in *d*$_6$-benzene.

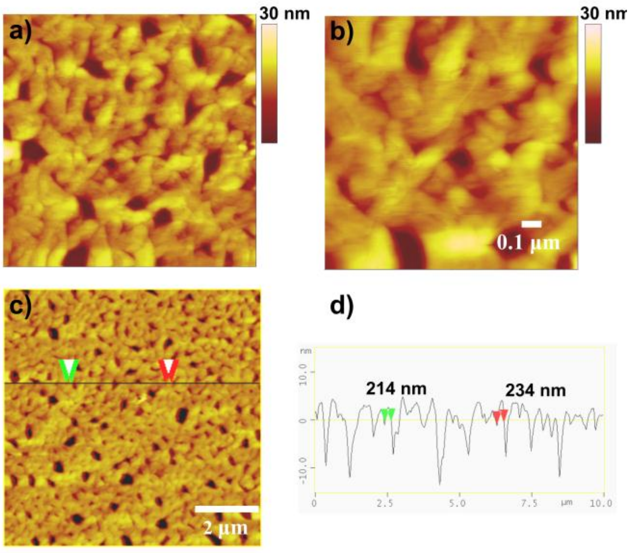

Figure S4: Additional AFM; (d) height image corresponding to (c).

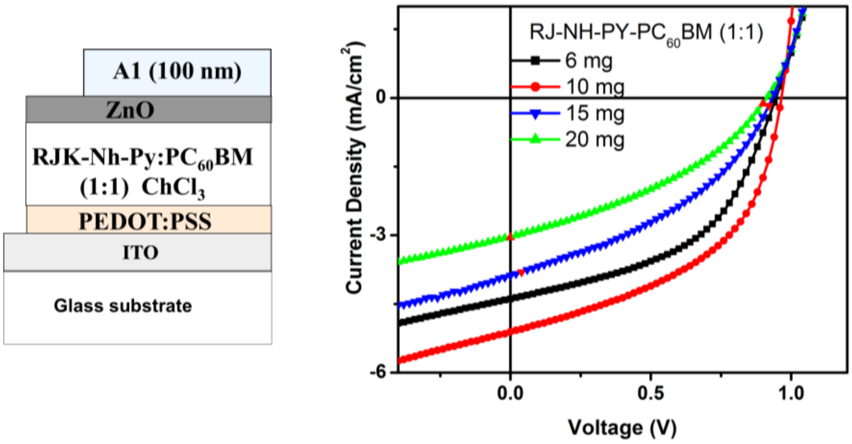

Figure S5: Current–voltage plots for compound **2**.

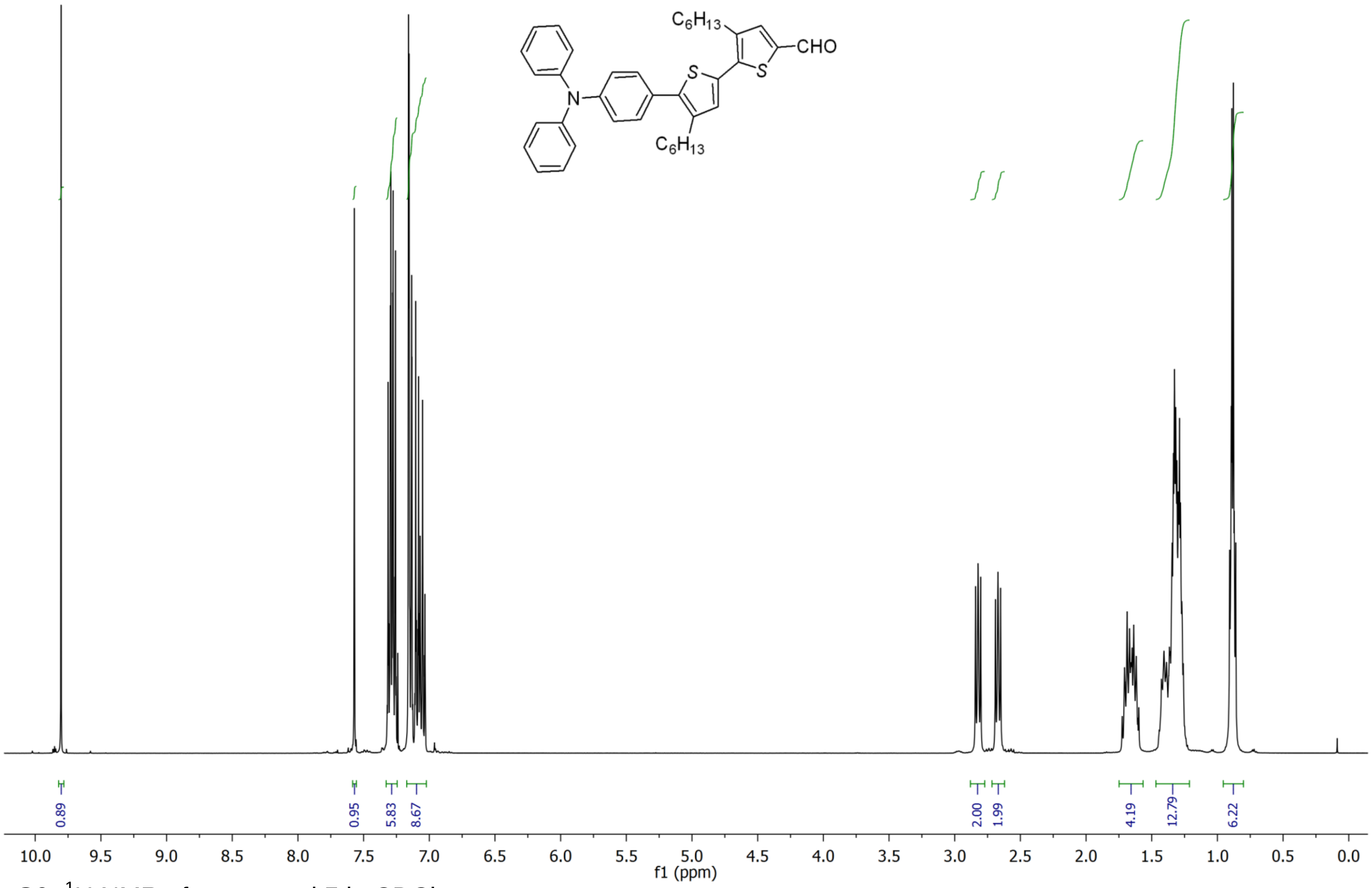

Figure S6: ^{1}H NMR of compound **5** in $CDCl_3$.

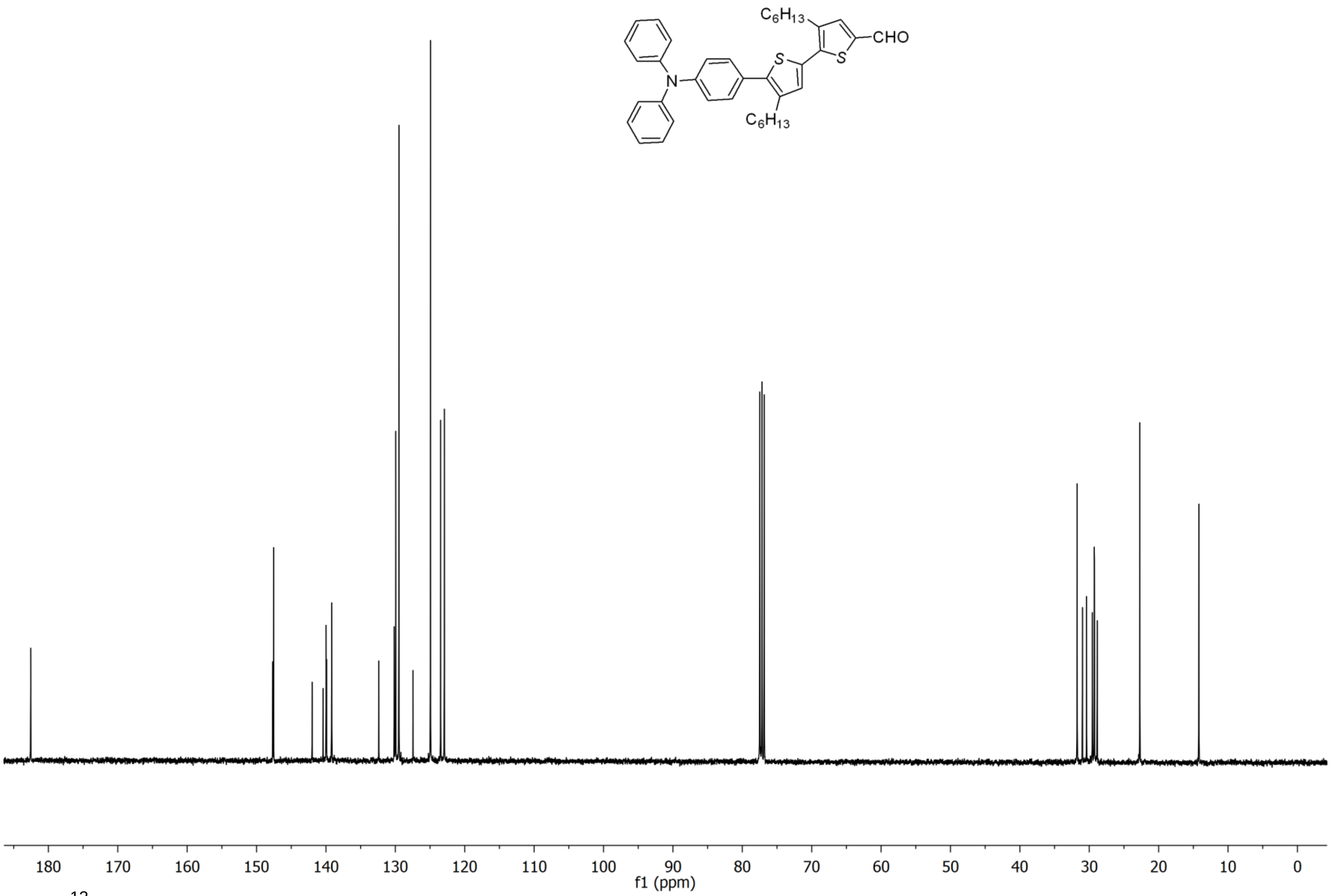

Figure S7: ^{13}C NMR of compound **5** in $CDCl_3$.

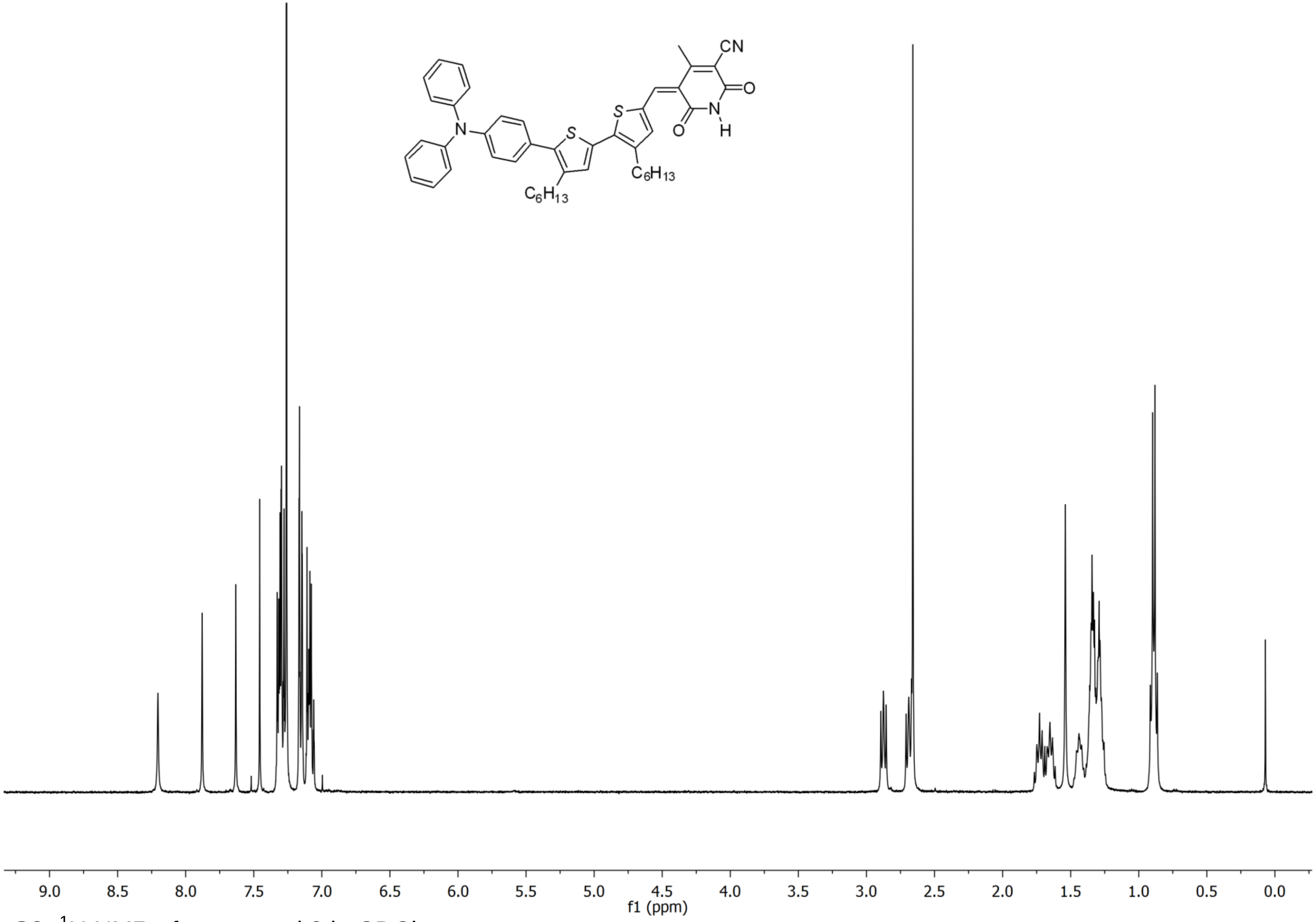

Figure S8: ^{1}H NMR of compound **2** in $CDCl_3$.

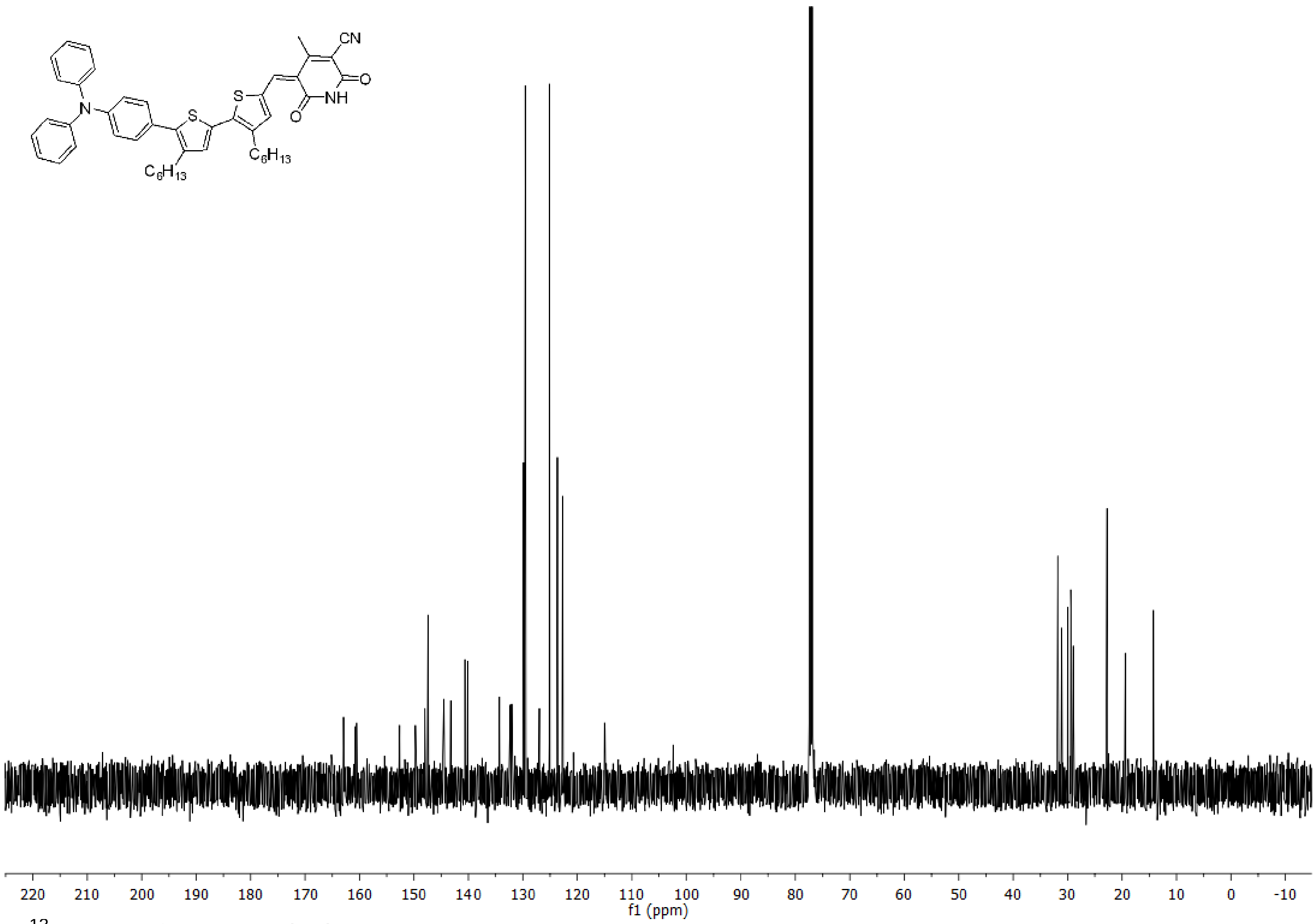

Figure S9: ^{13}C NMR of compound **2** in $CDCl_3$.

References

1. Ko, J. J.; Kim, D. H.; Choi, H. B. Novel compounds containing fused-triarylamine, methods of preparing the compounds and dye-sensitized solar cells using dyes comprising the compounds March 05, **2012**, KR 1109548, 64 pages.
2. Kiselev, E.; Agama, K.; Pommier, Y.; Cushman, M. *J. Med. Chem.* **2012**, *55*, 1682–1697. doi: 10.1021/jm201512x
3. Sarasqueta, G.; Choudhury, K. R.; Subbiah, J.; So, F. *Adv. Funct. Mater.* **2011**, *21*, 167–171. doi: 10.1002/adfm.201001328
